# Size and Shape-Dependent Antimicrobial Activities of Silver and Gold Nanoparticles: A Model Study as Potential Fungicides

**DOI:** 10.3390/molecules25112682

**Published:** 2020-06-09

**Authors:** Francis J. Osonga, Ali Akgul, Idris Yazgan, Ayfer Akgul, Gaddi B. Eshun, Laura Sakhaee, Omowunmi A. Sadik

**Affiliations:** 1 Sensors Mechanisms Research and Technology Center (The SMART Center), Chemistry and Environmental Science Department, New Jersey Institute of Technology, University Heights, 161 Warren Street, Newark, NJ 07102, USA; fosonga1@binghamton.edu (F.J.O.); gbe4@njit.edu (G.B.E.); 2 Department of Sustainable Bioproducts, College of Forest Resources, Mississippi State University, Starkville, MS 39759, USA; aa1116@msstate.edu; 3 Department of Chemistry, Center for Research in Advanced Sensing Technologies & Environmental Sustainability (CREATES), State University of New York at Binghamton, P.O. Box 6000 Binghamton, NY 13902, USA; iyazgan1@binghamton.edu (I.Y.); lsakhae1@binghamton.edu (L.S.); 4 Department of Basic Sciences, College of Veterinary Medicine, Mississippi State University, Starkville, MS 39759, USA; aa1625@msstate.edu

**Keywords:** luteolin, silver nanoparticles, luteolin tetraphosphate, gold nanoparticles, antimicrobial, remediation, fungicide, fungi

## Abstract

Plant-based pathogenic microbes hinder the yield and quality of food production. Plant diseases have caused an increase in food costs due to crop destruction. There is a need to develop novel methods that can target and mitigate pathogenic microbes. This study focuses on investigating the effects of luteolin tetraphosphate derived silver nanoparticles (LTP-AgNPs) and gold nanoparticles (LTP-AuNPs) as a therapeutic agent on the growth and expression of plant-based bacteria and fungi. In this study, the silver and gold nanoparticles were synthesized at room temperature using luteolin tetraphosphate (LTP) as the reducing and capping agents. The synthesis of LTP-AgNPs and LTP-AuNP was characterized by Transmission Electron Microscopy (TEM) and size distribution. The TEM images of both LTP-AgNPs and LTP-AuNPs showed different sizes and shapes (spherical, quasi-spherical, and cuboidal). The antimicrobial test was conducted using fungi: *Aspergillus nidulans*, *Trichaptum biforme, Penicillium italicum, Fusarium oxysporum,* and *Colletotrichum gloeosporioides,* while the class of bacteria employed include *Pseudomonas aeruginosa*, *Aeromonas hydrophila*, *Escherichia coli,* and *Citrobacter freundii* as Gram (−) bacteria, and *Listeria monocytogenes and Staphylococcus epidermidis* as Gram (+) bacterium. The antifungal study demonstrated the selective size and shape-dependent capabilities in which smaller sized spherical (9 nm) and quasi-spherical (21 nm) AgNPs exhibited 100% inhibition of the tested fungi and bacteria. The LTP-AgNPs exhibited a higher antimicrobial activity than LTP-AuNPs. We have demonstrated that smaller sized AgNPs showed excellent inhibition of *A. nidulans* growth compared to the larger size nanoparticles. These results suggest that LTP-AuNP and LTP-AgNPs could be used to address the detection and remediation of pathogenic fungi, respectively.

## 1. Introduction

The recent advances in nanotechnology are directed towards developing better techniques to control and eliminate diseases at the nanoscale level using engineered metal nanoparticles (MNPs) [1]. Antimicrobial activity of MNPs have received significant attention because nanoparticles possess a high surface-area-to-volume ratio and a platform to interact with microbes such as bacteria, viruses, and fungi. The rising cases of resistant bacteria strains due to antimicrobial drugs have led to the need to formulate drugs that can combat resistant bacteria and fungi [2,3,4,5]. 

Nanomaterials are promising candidates with numerous applications in diverse fields. Some metallic nanomaterials serve as bactericidal and bacteriostatic agents. The application of AuNPs is well-suited for biological systems due to their unique physio-chemical properties. It is worth noting that AuNPs possess the ability to undergo localized surface plasmon resonance (SPR), in the presence of light. As a result, a strong SPR band of AuNPs can be optimized to generate a color change [6]. This basis creates a platform for using AuNPs for microbial sensing, biocompatibility, and drug delivery studies [7]. The focus of utilizing AuNPs for controlling pathogenic microbes has received considerable efforts. Bindhu et al. reported the antibacterial activity of AuNPs against Gram-positive (*S. aureus*) and Gram-negative pathogens (*P. aeruginosa*) [8]. It was noted that the bactericidal effect of AuNPs possessed an appropriate smaller size as the platform for interacting with the microorganisms [6,7,8]. 

Furthermore, Chen et al. reported the effects of Au NPs for the size within the range of 8–37 nm and 3–100 nm on mice studies. It was revealed that the AuNPs with 8–37 nm were more toxic, while AuNPs with sizes between 3–100 nm were less toxic. The toxicity of AuNPs can be attributed to synthetic methods and the use of organic reducing and capping agents during the NP synthesis. It is clear that smaller sized AuNPs are more toxic due to the lower surface area [7].

Furthermore, nano-silver presents several advantages as antimicrobial agents. They possess a very high activity against a broad range of microbes. At lower doses, silver presents low toxicity toward humans and is relatively inexpensive [3]. Silver nanoparticles (AgNPs) have been reported as protective agents for cellular systems while inhibiting microbial growth. The antibacterial activity of nanoparticles has been reported, but their mode of action has been suggested. Several reports on the mechanistic mode of action of silver nanoparticles focusing on the effects of AgNPs has led to effective eradication of the target microbes. Liu et al. reported the possible mechanisms of AgNPs toxicity to bacteria by proposing that the AgNPs adhere to the bacteria surface and affect the membrane function [9]. The silver atom is inert and stable, but it becomes reactive when it assumes an oxidation state of +1. The silver ions (Ag^+^) bind to proteins in the plasma and nuclear membrane, forming a complex that leads to structural changes in the membrane [10,11]. Antimicrobial Ag^+^ are also released via the dissolution of AgNPs. The Ag^+^ ions react non-selectively with electron-donating groups such as thiols, hydroxyls, imidazoles, and phosphates [1]. AgNPs accumulate on the surface of the bacteria and form aggregates. As a result, the AgNP creates perforations in the cell wall, disrupting the integrity of the membrane, which leads to cell death [12].

AgNPs are also believed to exhibit antibacterial activity by triggering the formation of reactive oxygen species. These species then interact with the glycoproteins on the cell wall before being transferred into the cytoplasm, where they show major antibacterial activity [13]. Pal et al. reported a comparative report on how the shape of the AgNPs affects the activity against gram (−) bacterium, *E. coli*. Spherical AgNPs (Cubo- octahedral, or quasi-spherical shapes) (100) facets showed less antibacterial activity in comparison to the triangular (111) facets of AgNPs. Shape [14] and size [15] of the nanoparticles can make a difference even in the case of the nanoparticles possessing the same area-to-volume ratio. Apart from size and shape, surface modification and heterogeneity affect AgNPs’ antibacterial capability. A recent study reported that surface modification of AgNPs introduces a physical mechanism for antibacterial activity in addition to their inherent biological interference capability; silver-stearate NPs prevent the microbial attachments, including bacteria and fungi, to the food [13].

Flavonoid Luteolin (3′, 4′, 5, 7-tetrahydroxyflavone) is a glycosylated polyphenol, which is widely distributed in fruits and vegetables such as oranges, cabbages, and spinach. In literature, Luteolin is known to possess medicinal properties, which include its anti-inflammatory effects, antioxidant and anticancer effects against lung, skin, liver, and cervical cancers [15,16]. Luteolin also can inhibit proliferation and induced apoptosis of prostate cancer cells both in vitro and in vivo [15].

Nanoparticles have become a vital vehicle for the delivery of cancer therapeutics with high bioavailability, high efficacy, and improved permeability and retention effects. However, the solubility of Luteolin in water is poor, and this limitation has hindered the intravenous delivery of Luteolin. Also, Luteolin has previously been shown to alter the permeability of the bacterial cell wall without destroying it. Its antibacterial activity on *S. aureus* is believed to occur by blocking protein synthesis through interference with DNA enzymes such as DNA topoisomerase I and II [17]. Its molecular activity was also reported for amoxicillin-resistant *E. coli* because it can alter the membrane permeability and interfere with protein synthesis, thus limiting the required enzymes to fight against the noxious stimulus [18]. In this study, Luteolin was converted into a novel, water-soluble Luteolin tetraphosphate (LTP) derivative that was employed to synthesize the silver and gold nanoparticles as reported in our previous work [19].

The goal of this work is to investigate the detection and remediation of pathogenic fungi that lowers the yield of vegetable and fruit crops. Hence, this study revealed that when LTP is utilized as the reducing and capping agent in the synthesis of Ag and Au MNPs, the resulting Luteolin tetraphosphate (LTP)-mediated nanoparticles exhibit unique antifungal and anti-spores’ characteristics. We found that LTP- gold nanoparticles did not provide high antimicrobial activity. In contrast, the LTP-silver nanoparticles (LTP-AgNPs) showed a very promising shape and size-dependent antimicrobial activity against both Gram (−), gram (+) bacteria, and the fungi *A. nidulans*, *T. biforme*, *F. solani, P. italicum,* and *C. gloeosporioides*. 

## 2. Results

### 2.1. Synthesis and Characterization of LTP-Derived AgNPs and AuNPs 

**Figure 1:**
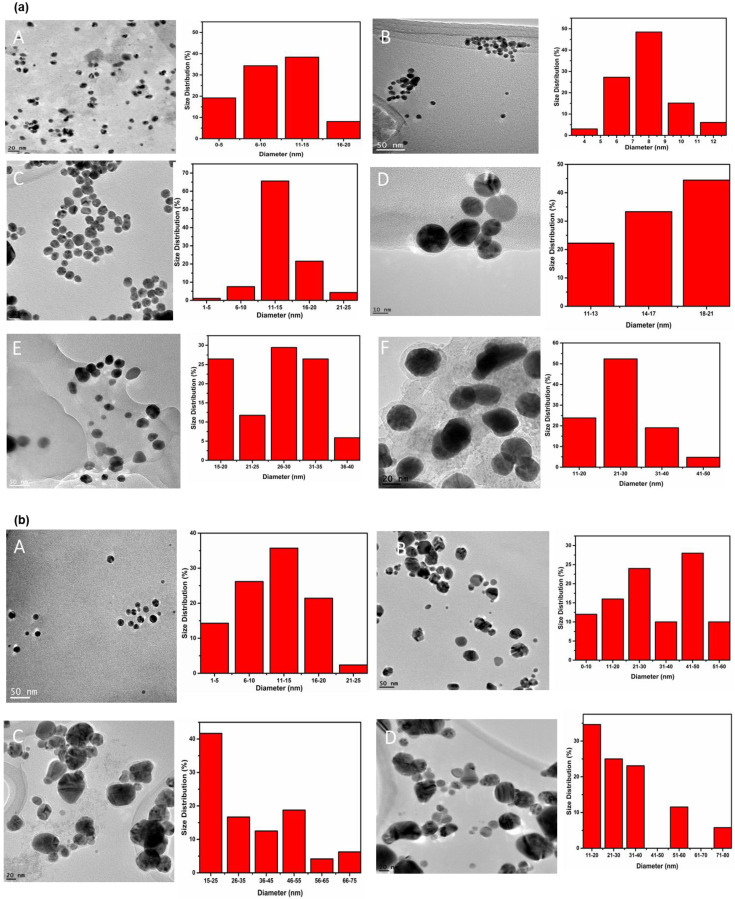
(**a**) TEM micrographs and corresponding histograms for luteolin tetraphosphate derived gold nanoparticles (LTP AuNPs) A (9nm); B (10 nm); C (15 nm); D (16 nm); E (26 nm); F (28 nm). (**b**) TEM micrographs and corresponding histograms for luteolin tetraphosphate derived silver nanoparticles (LTP-AgNPs) A (12 nm); B (30 nm); C (36 nm) and D (32 nm).

The synthesis and Physico-chemical characterization of AuNPs and AgNPs have been already reported in our previous work [19]. In this work, AuNPs and AgNPs were synthesized by using Luteolin tetraphosphate (LTP), which acted as a reducing and capping agent. The transmission electron microscopy (TEM) micrographs for AuNPs exhibited majorly spherical nanoparticles, as shown in Figure 1a (A–F). The TEM images and corresponding histogram show AuNPs of the size distribution of 9, 10, 15, 16, 26, and 28 nm. The role of LTP as a reducing agent and capping agent was demonstrated by the formation of dispersed AuNPs (Figure 1a) with sizes ranging from 9 to 28 nm, which were stable for eight months [19]. The synthesis of anisotropic nanoparticles (99% nanocubes) as reported in our previous work [19] was observed by reacting different concentrations of HAuCl_4_·3H_2_O with 600 μL of LTP at a mole ratio of 1:2; 2:3, and 5:6 (Figure 1a (E) and Figure S1D). The TEM images confirmed the formation of gold nanocubes (AuNCs), as shown in Figure 1 and Figure S1, with a size distribution of 16, 30, and 22 nm. TEM micrographs of LTP-AgNPs are presented in Figure 1b and Figure S1B,E,F) with a size distribution of 16, 20, and 22 nm. The TEM images revealed that the AgNPs were dispersed and were spherical and quasi-spherical in nature with an average diameter size of 12, 30, 36, 32 nm [Figure 1b (A–D)] and 9, 21, and 37 nm Figure S1B,E,F), respectively. 

**Figure 2:**
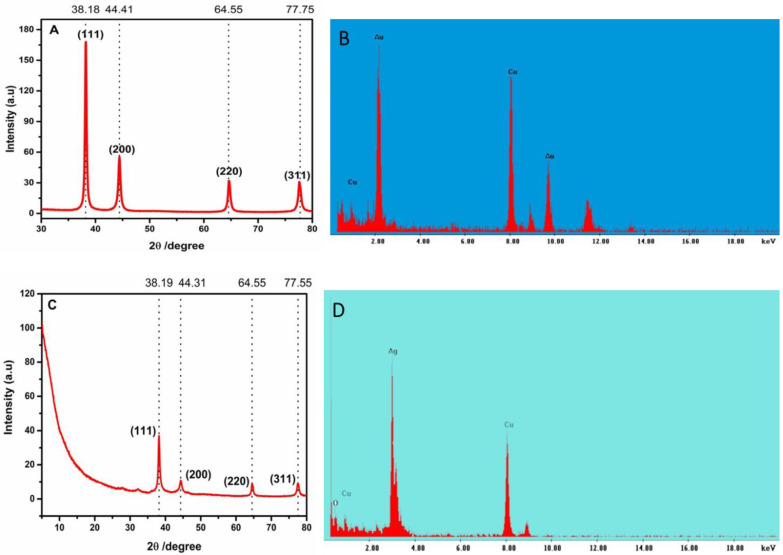
XRD patterns of AuNPs (**A**) and AgNPS (**C**); Energy-Dispersive X-ray Spectroscopy (EDX) spectrum of AuNPs and AgNPs (**B**) and (**D**), respectively.

Figure 2A shows XRD analysis of AuNPs depicting four characteristic diffraction peaks at 2θ = 38.19°, 44.46°, 64.60°, 77.75°, which could be indexed to (111), (200), (220), and (311) peaks representing characteristic diffraction of elemental metal Au^0^, which confirmed the formation of crystalline AuNPs ( JCPDS card No.04-0784) [20,21,22]. The EDX spectrum (Figure 2B) confirmed the formation of 100% elemental gold since copper originates from the TEM copper grid.

**Figure 3:**
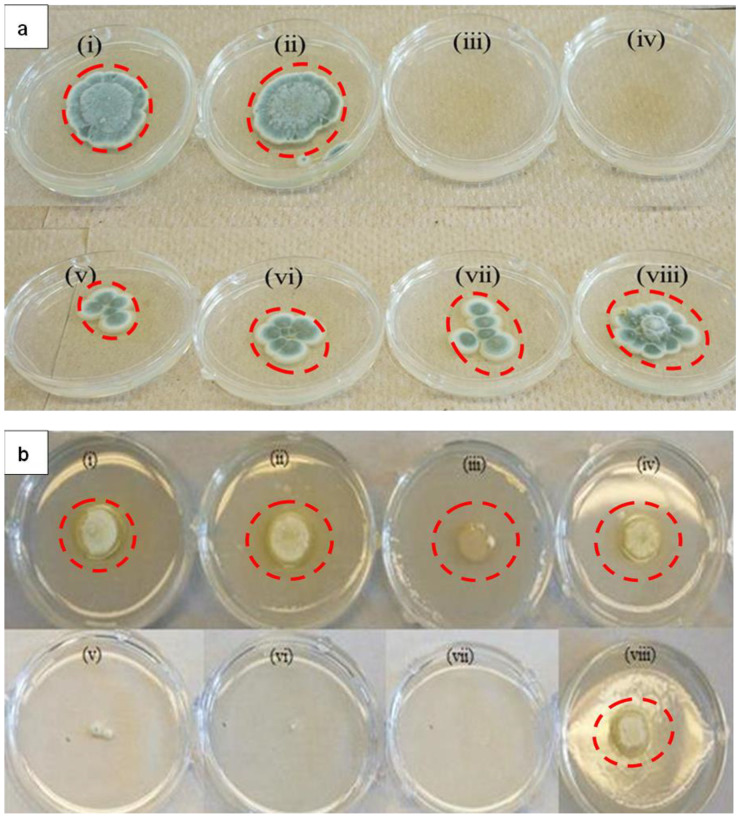
(**a**) Toxicity of 5 µM LTP-AgNPs on 10^3^ spores/mL *P. italicum* (i) control, (ii) LTP-AgNP4, (iii) LTP-AgNP1 (iv) LTP-AgNP5, (v) LTP-AgNP2, (vi) LTP-AgNP3, (vii) LTP-AgNP6 and (viii) LTP-AgNP7. LTP-AgNPs were filtered through 0.2 µm filter. LTP-AgNPs at 1, 5, 10 and 50 µL of the 5 µM concentrations were added to dextrose-tryptone broth media containing 10^3^ spores/mL *P. italicum*, which were incubated at 25 °C incubator for two-days. Then, 100 µL from each medium were transferred onto freshly prepared PDA agar, which were incubated 4-days at 25 °C incubator. (**b**) Toxicity of 5 µM LTP-AgNPs on *P. italicum* on PDA agar for 2 days incubation. LTP-AgNPs were filtered through 0.2 µm filter. 10^3^ spores in 100 µL were placed on the center of the plate, where 50 µL of the NPs were placed around the inoculum for (i), (ii), (iii) and (iv) for LTP-AgNP5, LTP-AgNP3, LTP-AgNP2 and LTP-AgNP7, respectively. While the NPs introduced to PDA agar right after the autoclave process (v), (vi), (vii) and (viii) for LTP-AgNP5, LTP-AgNP2, LTP-AgNP1 and LTP-AgNP7, respectively.

Figure 3C shows the results of XRD data exhibiting four characteristic diffraction peaks at 2θ = 38.19°, 44.46°, 64.60°, 77.75°, which could be indexed to (111), (200), (220), and (311) peaks representing characteristic diffraction elemental Ag^0^ and are consistent with standard data file JCPDS No. 04–0783 [23,24,25,26,27].

It is worth noting that in addition to spherical AgNPs, larger quasi-spherical AgNPs were also formed with an average diameter size of 21 and 37 nm, as shown in Figure S1E,F). Energy-Dispersive X-ray Spectroscopy (EDX) was performed on TEM in order to carry out an elemental analysis. The peaks of Cu normally occur since they are obtained from the TEM copper holding grid. The results show the formation of Ag atoms at 2.7 keV, thus EDX confirmed the formation of AgNPs [28,29,30]. The presence of oxygen could be due to oxygen species in flavonoids [29].

### 2.2. Toxicity of LTP-AgNPs on Fungi

LTP-AgNPs were screened to evaluate its toxicity effects on *P. italicum.* Two sets of LTP-AgNPs were used: suspended LTP-AgNPs and precipitated LTP-AgNPs. LTP-AgNPs were tested at 5 µM concentrations on *P. italicum*, and the results exhibited no difference between both the suspended and the precipitated LTP-AgNPs. The growth of fungi spores on agar plates with the treatment of suspended LTP-AgNPs are shown in Figure 3a. 

Table 1 shows the average diameter (mm) measurement representing the zone of inhibition for spores in each plate. The control in Figure 1a shows the greatest diameter since it did not receive the treatment of any LTP-AgNPs. The decrease in diameters of spores in each plates (v), (vi), (vii), (viii) after the treatment of various LTP-AgNPs was significant. Furthermore, none of the AgNPs showed 100% suppression of *P. italicum* growth at 1 µM concentration, while LTP-AgNP1 and LTP-AgNP5 suppressed the growth completely (Figure 1a). LTP-AgNP4 did not show any toxic effect at up to 50 µM at which concentration suppression was not significant. LTP-AgNP2, LTP-AgNP3, LTP-AgNP-6, LTP-AgNP7 prevented the growth of mycelia as depicted in Figure 3a (v), (vi), (vii), and (viii), respectively.

Figure 3b shows that the applied LTP-AgNPs did not make direct contact with the *P. italicum*. As a result, no significant toxicity was recognizable. However, the applied stress prevented the formation of a blue-green microscopic view of *P. italicum* for all of the tested LTP-AgNPs. In contrast to this, when LTP-AgNP5, LTP-AgNP2, and LTP-NP1 were evenly spread in PDA agar, dramatic suppression of *P. italicum* growth was observed. LTP-AgNP2 showed better anti-*P. italicum* capability on agar, which is unusual from Figure 3b (vi). In addition, the zone of inhibition measured was 10 mm, which was the same for the spores.

**Figure 4:**
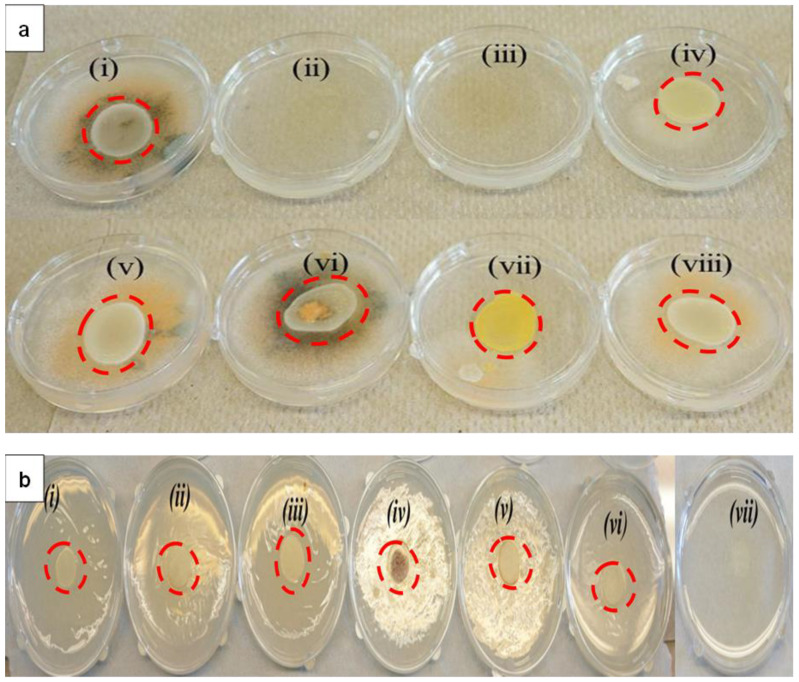
(**a**) Toxicity of 5 µM LTP-AgNPs on 10^3^ spores/mL *C.gloeosporioides* (i) Control, (ii) LTP-AgNP5, (iii) LTP-AgNP1, (iv) LTP-AgNP4, (v) LTP-AgNP2, (vi) LTP-AgNP3, (vii) LTP-AgNP6 and (viii) LTP-AgNP7. LTP-AgNPs were filtered through 0.2 µm filter. LTP-AgNPs were applied at 1–50 µM to 10^3^ spores/mL *C. gloeosporioides* in dextrose-tryptone broth for 2 days. Then, 100 µL from each medium were transferred onto freshly prepared PDA agar, which were incubated 4-days at 25 °C incubator. (**b**) Toxicity of 5µM LTP-AgNPs on *T. biforme* on PDA agar at 2-day incubation. LTP-AgNPs were filtered through 0.2 µm filter. 10^3^ spores in 100 µL were placed on the center of the plate, where the NPs introduced to PDA agar right after the autoclave process (i) *T. biforme* control, (ii) LTP-AgNP5, (iii) LTP-AgNP3, (iv) LTP-AgNP7, (v) LTP-AgNP6, (vi) LTP-AgNP1, and (vii) LTP-AgNP2.

Similar to *P. italicum*, *C. gloeosporioides* showed maximum vulnerability towards LTP-AgNP5 and LTP-AgNP1 (Figure 4a (ii)). LTP-AgNP4 (Figure 4a (iv)) and LTP-AgNP6 (Figure 4a (vii)) showed minimal effect while Figure 4a (v), (vi), and (viii) for LTP-AgNP2, LTP-AgNP3, LTP-AgNP7, respectively, showed no recognizable effect. Although LTP-AgNP2 showed recognizable toxicity towards *P. italicum*, it did not show any recognizable toxicity towards *C. gloeosporioides.* Silver nanoparticles, due to their strong anti-*C. gloeosporioides* capability, were offered as an alternative to fungicides [31]. The LTP-AgNP1 and AgNP5 provided stronger anti-*C. gloeosporioides* activity in comparison to the reported ones [31]. The diameter of the spores measured as 15 mm representing the zone of inhibition appeared to be similar for all the plates.

In contrast to *P. italicum* and *C*. *gloeosporioides*, *T. biforme* showed its maximum vulnerability towards LTP-AgNP2 (Figure 4 (vii)), followed by LTP-AgNP1 (Figure 4 (vi)).It is worth noting that *T. biforme* is a white-rotten mushroom, and this was the main difference of *T. biforme* from *P. italicum* and *C*. *gloeosporioides*. Interestingly, LTP-AgNP7 (Figure 4 (iv)) formed a shiny layer on the agar, which was not observed for *P. italicum* and *C*. *gloeosporioides*, and this could be attributed to the antimicrobial effect of the LTP-AgNP7. Finally, the diameter of spores in each plate was approximately 5 mm, the zone of inhibition was not significant. 

**Figure 5:**
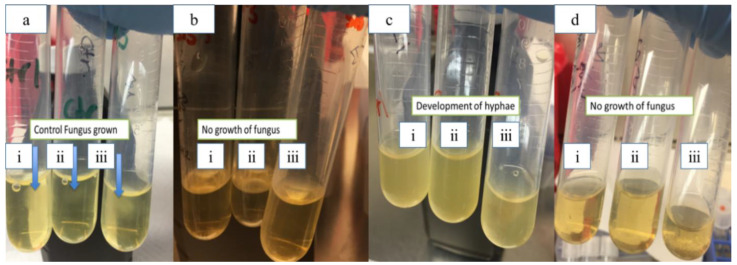
Turbidity study using *A. nidulans*: (**a**) Control of *A. nidulans*, (**b**) (i) 1 µM, (ii) 2 µM and (iii) 4 µM LTP-AgNP1, (**c**) (i) 70 µM, (ii) 140 µM and (iii) 280 µM LT, (**d**) (i) 10 µM, (ii) 72 µM and (iii) 144 µM LTP-AgNP5; 24 h incubation.

LTP-AgNP1 and LTP-AgNP5 were compared to the LT for their toxicities on *A. nidulans* (Figure 5 and Figure S2). At the tested concentrations, LTP-AgNP1 and LTP-AgNP5 showed complete suppression of *A. nidulans* while LT did not eliminate the growth even at 280 µM level (Figure 5). 

**Figure 6:**
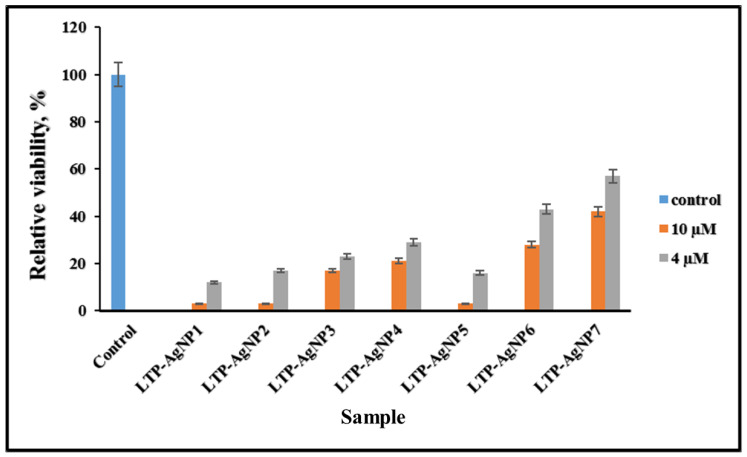
Toxicity of LTP-AgNPs on 10^3^ spores/mL *A. nidulans* in dextrose tryptone broth, incubated for 3 days at 25 °C.

When the incubation time was increased to 72 h, the growth of *A. nidulans* at 4 µM concentrations was observed. At 10 µM concentration, LTP-AgNP1, LTP-AgN2, and LTP-AgNP5 completely suppressed the growth (Figure 6). 

### 2.3. Toxicity of LTP-AgNPs on Bacteria

**Figure 7:**
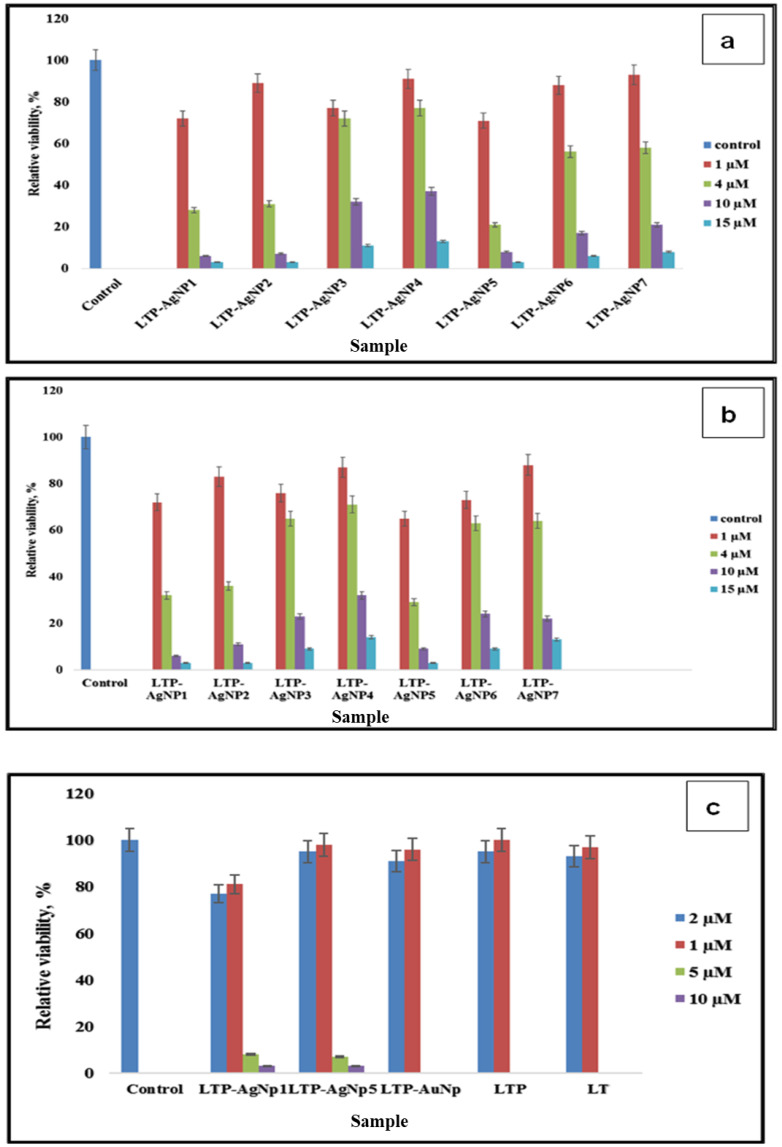
(**a**) Toxicity of LTP-AgNPs on 10^4^ cfu/mL *Staphylococcus epidermidis* in nutrient broth. At 15 µM, the absorbance of LTP-AgNP1 and LTP-AgNP5 did not give a higher value than the initially measured one, so to make it visible, relative viability was assigned as 3. (**b**) Toxicity of LTP-AgNPs on 10^4^ cfu/mL *Citrobacter freundii* in nutrient broth. At 15 µM, the absorbance of LTP-AgNP1 and LTP-AgNP5 did not give a higher value than the initially measured one, so to make it visible, relative viability was assigned as 3. (**c**) Toxicity of LTP-NPs on 10^4^ cfu/mL *Escherichia coli* in nutrient broth.

**Figure 8:**
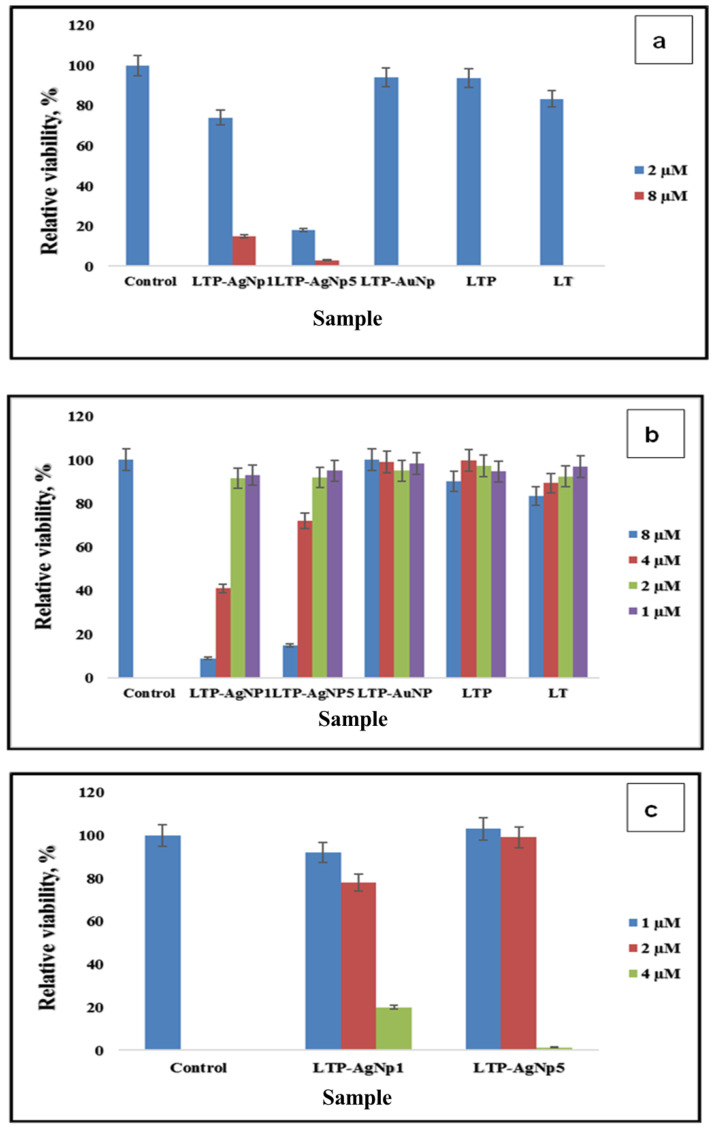
(**a**) Toxicity of LTP-NPs, LTP, LT on 10^4^ cfu/mL *Aeromonas hydrophilia* in lysogeny broth. (**b**) Toxicity of LTP-NPs, LTP, LT on 10^4^ cfu/mL *Pseudomonas aeruginosa* in lysogeny broth. (**c**) Toxicity of LTP-AgNPs on 10^4^ cfu/mL *Listeria monocytogenes* in lysogeny broth.

LTP-AgNPs showed toxicity towards all the tested bacterial cultures (Figure 7a–c; Figure 8a–c; Figures S3 and S4). The vulnerability of the bacteria towards the LTP-AgNPs did not show dependence on the used LTP-AgNPs; instead, the vulnerability was dependent on the concentration of the AgNPs. Among the tested LTP-AgNPs, only LTP-AgNP1 and LTP-AgNP5 showed complete suppression of the growth at the tested highest levels, 15 µM. 

Similar to antifungal capability, LT, LTP, and LTP-AuNPs did not show strong antibacterial capability, while corresponding LTP-AgNPs showed promising antibacterial ability. 

**Figure 9:**
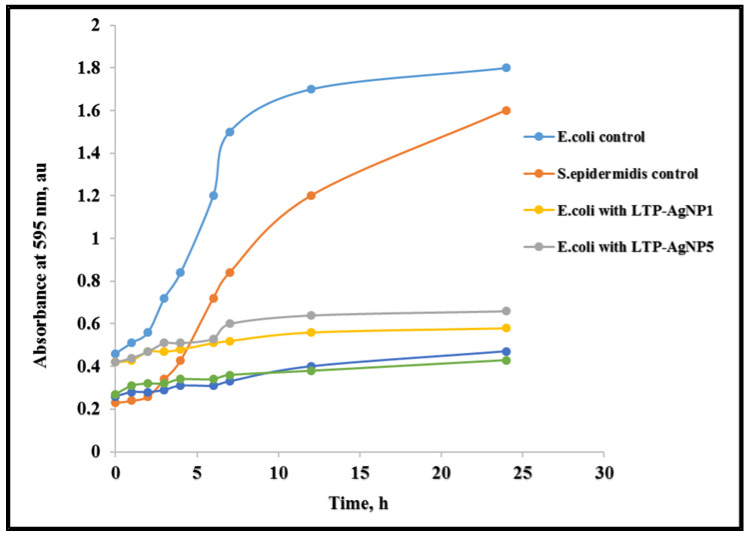
Effect of 10 µM LTP-AgNP1 and 10 µM LTP-AgNP5 on the growth kinetics of *E. coli* and *S. epidermidis* in Mueller-Hinton Broth.

To investigate the inhibitory effects of LTP-AgNPs on the growth of bacterial culture, we treated *E. coli* with 10 µm LTP-AgNP1 for the initial reaction. We treated the next *E. coli* culture with 10 µm LTP-AgNP5, and the optical density at 595 nm (OD_595_) of the resulting bacterial cultures was measured at 1 h time intervals. Figure 9 shows the measurements of OD at 595 nm taken over an hourly time interval for 25 h. 

The OD_595_ values were as follows: 0.43 for *E. coli* (control), 0.24 for *S. epidermidis* (control). After the initial 4 h growth mark, the OD_595_ for *E. coli* when treated with LTP-AgNP1 and LTP-AgNP5 were approximately 0.42, while the OD for the control was around 0.9 a.u. We observed that the control set-up grew exponentially between 9–10 h, while the growth rate of treated bacterial culture plateaued around 10 h. This pronounced difference was expected since 10 µm LTP-AgNP1 and 10 µm LTP-AgNP5 inhibited the growth of the bacterial culture. 

Furthermore, to explain how the LTP-AgNPs showed their antibacterial capability, growth kinetics of *E. coli* and *S. epidermidis* underexposure of LTP-AgNP1 and LTP-AgNP5 were tested. Under no stress conditions, both *E. coli* and *S. epidermidis* entered the log phase, where their growth was exponential. On the contrary, both *E. coli* and *S. epidermidis* could not surpass the stress introduced by the AgNPs to boost-up their growth. Therefore, it can be speculated that the lethal dose of LTP-AgNPs confined to the tested bacteria into lag-phase, where no actual overall growth can be claimed. This is a common observation for the toxic levels of AgNPs [13,32]. Furthermore, the dissolution of LTP-AgNPs silver releases antimicrobial silver ions that interact with thiol functional groups contained in the proteins embedded in the cell wall. This alters the structural function and the growth of the bacteria. This is the proposed mechanism underlying the antimicrobial activity for silver nanoparticles [33].

### 2.4. Cytotoxicity of LTP-AgNPs

**Figure 10:**
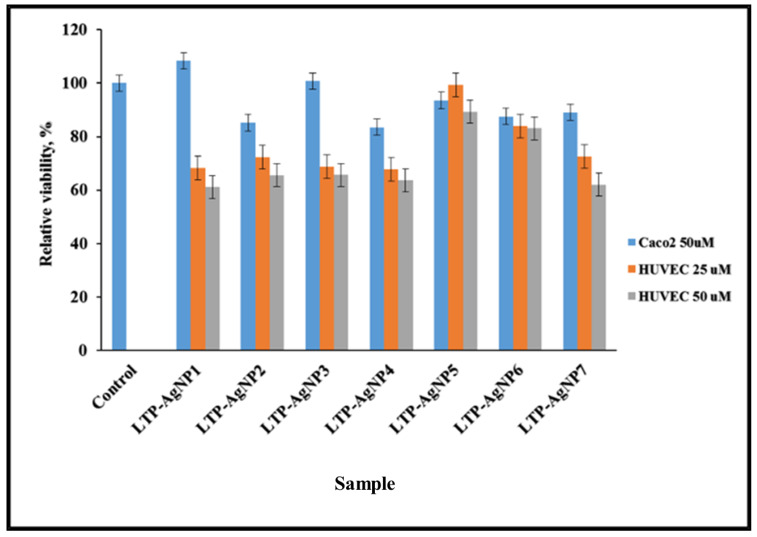
Cytotoxicity of LTP-AgNPs on Caco-2 and HUVEC cells.

The Caco2 cell line originates from human colon carcinoma. It serves as a model that mimics the in vivo system by exhibiting specialized enterocytes function via biochemical activities as the result of the enzymes located in the small intestines [34]. They are heterogeneous and possess an ability to differentiate spontaneously to express different morphologies and functions, which are features of matured enterocytes [35]. HUVEC cells are models obtained from the endothelium of veins located in the umbilical cord. Caco-2 cells and HUVEC cells are both primary epithelial cell lines. The NPs exposure was 24 h since, for the first ~10h, the toxicity might not be clearly seen [36], which could provide biased results. All the LTP-AgNPs showed toxicity towards the cell lines non-selectively. Since HUVEC cells are more delicate, they showed higher vulnerability, which was concentration-dependent. It should be noted that the tested concentrations (i.e., 25 and 50 µM) completely suppressed the growth of the tested cell lines. However, the least viable cell line (HUVEC 50 uM) was approximately 60% (Figure 10). 

## 3. Discussion

The tested LTP-AgNPs showed promising antimicrobial capabilities against both the tested fungi and bacteria, while their cytotoxic effects were relatively limited. The antifungal capacity of the tested AgNPs was higher in comparison to those obtained against the tested bacteria. However, in all cases, the size and shape of the LTP-AgNPs brought the antimicrobial capability. In contrast to this, LTP-AuNPs and LTP and LT compounds did not show any comparable antimicrobial activity in comparison to LTP-AgNPs. 

Here, it can be speculated that silver nanoparticles generate silver ions during incubation while gold nanoparticles were more strictly stabilized by LTP with a limited release of ions into the media. AgNPs enter the cell via receptor-mediated endocytosis. Following a cascade pathway, the silver nanoparticles initially end up in the endosome and then continue to the lysosome. AgNPs faces a varying acidic environment of pH 4 to pH 5. Liu has reported that at lower pH, there is an increase in the ion release while investigating the dissolution of silver nanoparticles at different pH [37]. 

In that respect, smaller size AgNPs exhibited greater antimicrobial activity compared to the larger ones. The smaller size AgNPs accumulates easily and has a greater effect on the target organelle in comparison to the larger size nanoparticles [38]. Studies have shown that the size of AgNPs plays a significant role in membrane disruption of microbes and hence affects the bacteria viability [33,39,40,41,42,43,44].

The antifungal activities of silver nanoparticles have been reported for a variety of fungi such as humans [44,45], plants [46], and household pathogens [47]. Therefore, mechanistic studies have been conducted here to further understand the activity of AgNPs towards fungi. AgNPs can enter the fungi by damaging cell membranes and cell surface, which then disrupts organelles such as the energy center (mitochondria) and essential units of protein synthesis (ribosomes) [48]. In the case of small-sized nanoparticles, the possibility of this event could be significant because these would not require any significant disruption of the cellular membrane. Therefore, we believe that the small-sized spherical nature of LTP-AgNP1 enhanced antimicrobial capability. In agreement with prior work, this observation could be attributed to the available area-per-volume that interacts with the cellular membrane of *A. nidulans* [44]. 

The results clearly depict that at 5 µM concentrations, LTP-AgNP1 (9 nm spherical) and LTP-AgNP5 (quasi-spherical 21 nm) showed 100% suppression of growth of *P. italicum* (Figure 3) and *C. gloeosporioides* (Figure 4a) for the tested 4-day incubation period. LTP-AgNP6 (37 nm quasi-spherical) inhibited 60% growth of *P. italicum* (Figure 3) and 70% mycelial growth of *C. gleoesporoides* (Figure 4a) with altering the macroscopic view. Although LTP-AgNP2 and LTP-AgNP3 inhibited ~50% growth of *P. italicum,* they did not show any effect on *C. gloeosporioides*. The same trend was observed for *A. nidulans*; LTP-AgNP1, LTP-AgNP2, and LTP-AgNP5 showed a similar capability to inhibit growth while LTP-AgNP7 showed nearly no effect (Figure 6). However, LTP-AgNP7 (21 nm spherical) did not show any strong impact on both *P. italicum* and *C. gloeosporioides*, while it was observed the only AgNP inhibited the growth of *T. biforme*. Pal et al. (2007) reported the shape is critical in revealing the toxicity of the AgNPs [13]; the quasi-spherical AgNPs with their truncated nature might have found strong interaction capability with the fungal cells. These two plant pathogens are among the most common post-harvest disease triggering fungi, so the outstanding antifungal ability of the AgNPs can make them attractive as an alternative to fungicides. 

The tested LTP-AgNPs showed a unique impact on mycelial growth of *P. italicum,* which was not observed for the rest of the fungi. As seen from Figure 3a, LTP-AgNP2, LTP-AgNP3, LTP-AgNP6, and LTP-AgNP7 prevented one unit-body mycelial formation. This could be related to the fact that LTP-AgNPs blocked the continuous growth of mycelia through distorting the hyphae wall [31], the cell surfaces, and interfering with the signaling cascades [49]. *P. italicum* was shown to be capable of synthesizing AgNPs [50] as well, which could also be one of the possibilities of the altered response of *P. italicum* to the LTP-AgNPs.

Previous studies have shown that AgNPs derived from phosphorylated flavonoids exhibited excellent antibacterial activity [25,51]. In addition to size, the shape also showed the importance of similar-sized LTP-AgNPs, where quasi-spherical LTP-AgNPs showed higher capability (LTP-AgNP5) than corresponding similar-sized spherical ones. The presented concentrations for antifungal and antibacterial are only the ones in which one of the tested LTP-AgNPs gave 100% suppression for the tested fungi and bacteria. Therefore, 4–10 µM for antifungal tests and 2–15 µM for antibacterial tests are the accepted highest concentrations to be presented in the study.

The antibacterial capability of LTP-AgNPs showed a similar trend observed for the tested fungi. LTP-AgNP1 and LTP-AgNP5 showed the best antibacterial activities towards the tested bacterial species, for which 4–15 µM concentrations range were observed as the minimal concentration to inhibit 100% growth. In contrast to the fungi, size effect was more evident for the antibacterial capability; LTP-AgNP2 (16 nm spherical) showed unique antibacterial capability, which was not seen observed as an excellent antifungal agent. Similarly, at 15 µM, the rest of the LTP-AgNPs eliminated over 70% of *C. freundii* and *S. epidermidis*. The observed results for *A. hydrophilia*, *P. aeruginosa*, *E. coli,* and *L. monocytogenes* were slightly different than *C. freundii* and *S. epidermidis* for the lowest concentration to inhibit over 99.99% growth (Figure 7a–c and Figure 8a–c). Furthermore, at 8 µM LTP-AgNP1 only eliminated ~70% of the growth, which did not follow the commonly observed trend for the tested microorganisms. *A. hydrophilia*, *P. aeruginosa*, and *L. monocytogenes* are pathogenic bacteria, which require intensive antibiotic treatment because of their antibiotic-resistant natures. The developed AgNPs showed very high toxicity towards them, which can open a new window for these relatively benign AgNPs to be used as antibiotics. 

LTP-AgNP5 (21 nm quasi-spherical) showed similar capability to LTP-AgNP1 (9 nm spherical) and higher potential than LTP-AgNP2 (16 nm spherical). For similar-sized LTP-AgNPs, shape brought dramatic effect, where LTP-AgNP2, in general, required at least two-times concentration of LTP-AgNP5 to show the same effect on tested fungi and bacteria. However, interestingly LTP-AgNP7 (21 nm spherical) did not show any descent antimicrobial activity except towards *T. biforme*. This is a dramatic observation that shifting from quasi-spherical to spherical at 21 nm size; the antimicrobial capability was not observable the presented concentrations. The antifungal capability of the tested LTP-AgNPs obtained was higher in comparison to those obtained against the tested bacteria. Although the antimicrobial activity of LTP-AgNPs and LTP-AuNPs were effective at sizes below 100 nm, the cubic shaped LTP-AuNPs at sizes 372 and 510 nm did not exhibit any antimicrobial activity. This is in agreement with previous studies which demonstrated that smaller nanoparticle sizes exhibited significant antimicrobial activity.

Future work will focus on the treatment of wastewater by AgNPs to destroy resistant bacteria.

## 4. Materials and Methods 

### 4.1. Synthesis and Physicochemical Characterization of Gold and Silver Nanoparticles 

The synthesis of LTP followed the procedure described in our previous work [19,52] with slight modification in the use of equivalents of Dibenzyl phosphite (Sigma-Aldrich, St. Louis, MO) in phosphorylation of Luteolin (Indofine Chemicals inc., Hillsborough, NJ). Flash chromatography Combiflash companion/TS Model serial 207L20329, Teledyne Isco, Inc., was used for the purification of the products. ^1^H, ^13^C, ^31^P NMR spectra were obtained using 600 MHz (Bruker Avance). The TLC analyses were performed using 0.25 mm EM Silica Gel 60 F250 plates visualized by UV irradiation (254 nm). 

Synthesis and physicochemical characterization of AgNPs and AuNPs were already described as reported in our previous work [19]. Typically, in this study, AgNPs and AuNPs were synthesized by using Luteolin tetraphosphate (LTP) as the reducing and stabilizing agent, as reported in our previous work [15]. Briefly, AuNPs were synthesized by reacting 300 μL of 5 mM gold (III) chloride (HAuCl_4_·3H_2_O, Sigma-Aldrich, Milwaukee, WI) with different concentrations of 5 mM LTP whereby 200, 300, 400, 600, and 800 μL LTP for the formation of spherical and quasi-spherical nanoparticles. The second set was done by varying the concentration of HAuCl_4_·3H_2_O whereby 100, 200, 300, 400, and 500 μL of 5 mM HAuCl_4_·3H_2_O were each reacted for 90 min with 600 μL of 5 mM LTP for the formation of gold nanocubes. The AuNPs were washed three times using nanopure water. In this synthesis, 4.5 mM of silver nitrate (AgNO_3_, 99%, Sigma-Aldrich, Milwaukee, WI) was reacted with different concentrations of L.T.P. AgNPs were synthesized by varying the concentration of 5 mM AgNO_3_: 5 mM LTP in the ratio of 1:1, 1:2, 1:3, 2:1.5, and 2:3. The reaction was carried out at room temperature and monitored using UV-Vis for about 300 min. The AuNPs and AgNPs solutions formed were sonicated for 30 min in an ultrasound bath and were then centrifuged 3500 rpm for 30 min to obtain pellets, which were then washed three times using 18 MΩ cm nanopure water. UV/Vis absorption spectra were carried out on an HP 8453 UV-visible diode array spectrophotometer. 18 MΩ cm nanopure water was used in the preparation of reagents. XRD analysis was done using Bruker D8 Advance 800234-X-ray (9729) at 40 kV and 40 mA. Transmission electron microscopy (TEM) studies of AuNPs and AgNPs were performed on a JEOL TEM 2100F. The TEM experiments were conducted by adding a drop of the samples to the carbon-coated copper grid (Sigma-Aldrich, Milwaukee, WI) and then dried. 

Seven different sizes and shapes of LTP-AgNPs were utilized for the antimicrobial and cytotoxic activities. The nanoparticles used include, LTP-AgNP1 (spherical, 9 nm), LTP-AgNP2 (spherical, 16 nm), LTP-AgNP3 (spherical, 30 nm), LTP-AgNP4 (spherical, 35 nm), LTP-AgNP5 (quasi-spherical, 21 nm), LTP-AgNP6 (quasi-spherical, 37 nm) and LTP-AgNP7 (spherical, 21 nm). The LTP-AuNPs were also synthesized by using LTP. The sizes and shapes of LTP-AuNPs were recorded and determined as follows: LTP-AuNP1 (spherical, 8 nm)**,** LTP-AuNP2 (spherical, 9 nm), LTP-AuNP3 (spherical, 10 nm), LTP-AuNP4 (cubic, 16 nm), LTP-AuNP5 (cubic, 20 nm), LTP-AuNP6 (cubic, 372 nm) and LTP-AuNP7 (cubic, 510 nm). The representative size distribution of the AuNPs and AgNPs are presented in Figure 1, Figure 2, and Figure S1. Furthermore, the summary of the shapes and sizes of the synthesized AuNPs and AgNPs are presented in Table 2.

Different concentrations of LTP-AgNPs and AuNPs ranging from 1 µM to 280 µM were prepared by dissolving the nanoparticles in nanopure water. Both LTP-AgNPs and LTP-AuNPs were compared to the LTP, LT, and LTP-AuNPs. The nanoparticles were tested for their antifungal activities in two ways: (i) precipitated and (ii) suspended forms. In the precipitated types, the precipitated AgNPs and suspended ones were utilized together while the suspended were the ones were obtained by filtering the stock AgNPs solution with 0.2 µm filter, which eliminated any visible particles in the AgNPs solution. Only suspended AgNPs were utilized for the antibacterial and cytotoxicity tests.

### 4.2. Antifungal Studies

#### Turbidity/Agar Studies

*Aspergillus nidulans* ATCC^®^ and *Trichaptum biforme* ATCC^®^ were grown in peptone-yeast media while *Penicillium italicum* ATCC^®^ and *Colletotrichum gloeosporioides* ATCC^®^ were grown in dextrose-tryptone broth. A total 10^3^ spores were inoculated into 2–3 mL of fresh liquid media. A total of 30 mg/mL Yeast Peptone Dextrose (YPD) medium was autoclaved before the introduction of nanoparticles followed by *A. nidulans* or *T. biforme*. The preparation of liquid media made of dextrose-tryptone broth was conducted by following the manufacturer’s procedure. The media containing fungi were vortexed at 8 h intervals to prevent any hyphae formation, and the incubation was performed in 25 °C incubator for 48 h. The turbidity of fungi was measured at 600 nm via HP 8453 UV-Vis spectrometry. In the case of solid culture, the fungal cells were performed on Nutrient Agar.

### 4.3. Antibacterial Studies

#### 4.3.1. Turbidity Studies

The tested microorganisms were *Pseudomonas aeruginosa* ATCC^®^, *Aeromonas hydrophila* ATCC^®^, *Escherichia coli* ATCC^®,^ and *Citrobacter freundii* ATCC^®^ as Gram (−) bacteria, and *Listeria monocytogenes* ATCC^®^ and *Staphylococcus epidermidis* ATCC^®^ as Gram (+) bacteria. Lysogeny broth (LB), a nutritionally rich medium, was used for the maintenance of the tested *P. aeruginosa*, *A. hydrophilia*, and *L. monocytogenes* while nutrient broth medium was used for *E. coli*, *C. freundii*, and *S. epidermidis,* respectively. All tests were repeated 2 times by 3 replicates of plates. All the microorganisms were propagated using broth media, and then nanoparticles were exposed to the organisms in their log phase. Incubation was carried out at 37 °C for 18 h. To prevent the microbial growth inhibited by nutrient scarcity, initial bacterial was kept at 10^3^ cfu/mL; the observed growth inhibition was attributed to the presence of the nanoparticles. After 18 h incubation, UV-Vis was used to measure the optical density at 595 nm for the control and nanoparticle treated bacterial cultures. This was achieved by diluting the media twice with 50 mM phosphate buffer saline at pH 7.4

The experiments dealing with *A. nidulans*, *T. biforme*, *P. italicum*, *C. gloeosporioides* were performed in biosafety level 2 laboratory facilities located in the Department of Psychology at SUNY-Binghamton while *L. monocytogenes*, *A. hydrophilia*, and *P*. *aeruginosa* were conducted in the Department of Sustainable Bioproducts, College of Forest Resources and the Department of Basic Science in the College of Veterinary Medicine at Mississippi State University. 

#### 4.3.2. Bacterial Growth Kinetics in Response to Nanoparticle Treatment

The LTP-AgNPs showed the best antibacterial activity was utilized at 10 µM concentrations in order to monitor *E. coli* and *S. epidermidis* growth kinetics. Mueller Hinton Broth containing *E. coli* and *S. epidermidis* concentrations of 10^3^ cfu/mL were monitored for 24 h. The OD_600_ measurements for both the *E. coli* and *S. epidermidis* were recorded and depicted as a graph in the results section.

### 4.4. Cytotoxicity of the LTP-AgNPs

Colon cancer cell line (ATCC^®^ Caco-2 cells) and primary epithelial cell line (ATCC^®^ 1730 HUVEC cells) were used to evaluate the cytotoxicity of the LTP-AgNPs. Caco-2 cells were cultivated in Eagle-Modified-Essential-Medium containing 0.01 u/g insulin, 0.02% L-glutamine, 0.02% non-essential amino acids and 9% Fetal-bovine serum albumin. In contrast, HUVEC cells were cultured in Endothelial Cell Growth Media (Sigma-Aldrich, Cat. 211-500), which was used as purchased from the vendor.

A total of 150 µL of 24 h incubated cells were plated in 96-well plate at 37 °C, 5% CO_2_ incubator, in which the initial confluence was 30%, were treated with 50 µM of the filtered LTP-AgNPs for 24 h. A total of 9 µL of PrestoBlue® fluorescent dye (Thermofisher, A13261) was added to each well and incubated at 37 °C for 20 min, which was then read with Biotek HT microplate-reader where excitation and emission wavelengths were 595 nm and 645 nm, respectively.

## 5. Conclusions

This study focused on the size effect antimicrobial activity of LTP-AgNPs and LTP-AuNPs. The TEM and size distribution characterization of LTP-AgNPs and LTP-AuNPs showed different sizes and shapes. The antimicrobial activity of LTP-AgNPs and LTP-AuNPs against selected plant-based pathogens demonstrated that smaller sized LTP-AgNPs had the strongest antimicrobial activity. From these results, the LTP derived AgNPs have the potential to be used as antimicrobial agents. Furthermore, the low cytotoxicity of the colloidal LTP-AgNPs confirms that LTP-AgNPs could be applied as an antimicrobial remedy for infections caused by plant pathogens such as *P. italicum* and *C. gloeosporioides*. The antibacterial results revealed that the LTP-AgNPs inhibited the growth of both Gram (+) and Gram (−) bacteria; however, the inhibition was greater for Gram (+) bacteria. This study emphasizes cost-effective and eco-friendly antimicrobial agents as an alternative to conventionally used antimicrobial agents. LTP-AgNPs and LTP-AuNPs materials can find applications in remediation, drug delivery, and treatment against other pathogens.

## Figures and Tables

**Table 1:** Average diameter measurement of spores showing effects of LTP-AgNPs on the growth of *P. italicum.*

Types of NP	Day 2 (mm)	Day 4 (mm)
Control	12.5	18
LTP-AgNP1	0	0
LTP-AgNP2	8	13
LTP-AgNP3	9	14
LTP-AgNP4	12	17
LTP-AgNP5	0	0
LTP-AgNP6	7	12
LTP-AgNP7	11	15

**Table 2:** Summary of shapes and sizes of synthesized LTP-AgNPs and LTP-AuNPs.

Parameters of NP	LTP- AgNPs		LTP-AuNPs	
Shape	Spherical	Quasi-spherical	Spherical	Cubic
Sizes (nm)	9, 16, 30, 35	21, 37, 21	8, 9, 10	16, 20, 372, 510

## Supplementary Materials

Figure S1: Histograms and the corresponding TEM micrographs (inset) for (A) LTP-AuNP1 (spherical, 8 nm), (B) LTP-AgNP2 (spherical, 16 nm), (C) LTP-AuNP3 (spherical, 10 nm), (D) LTP-AuNP4 (cubic, 16 nm), E) LTP-AgNP5 (quasi-spherical, 21 nm), (F) LTP-AgNP6 (quasi-spherical, 37 nm), Figure S2: (a) Control of *A. nidulans*, (b) (i) 1 µM, (ii) 2 µM and (iii) 4 µM LTP-AgNP1, (c) (i) 20 µg/mL, (ii) 40 µg/mL and (iii) 80 µg/mL LTP, (d) (i) 36 µM, (ii) 72 µM and (iii) 144 µM LTP-AgNP2 after one week growth, Figure S3: (a) 36 µM LTP-AgNP2 treated *P. aeruginosa*, (b) 18 µM LTP-AgNP2 treated *A. hydrophila*, Figure S4: (a) 4 µM LTP-AgNP1 treated *A. hydrophila* and (b) (i) 4.5 µM, (ii) 9 µM and (iii) 18 µM LTP-AgNP2 treated *L. monocytogenes* samples.
