# Supplementary material for: Size and Shape-Dependent Antimicrobial Activities of Silver and Gold Nanoparticles: A Model Study as Potential Fungicides
Source: Molecules. 2020 Jun 9;25(11):2682. doi: 10.3390/molecules25112682 (PMC7321160; doi:10.3390/molecules25112682)
Supplement: Supplementary file 1 [file molecules-25-02682-s001.pdf]

Supporting Information

Size and Shape-Dependent Antimicrobial Activities of Silver and Gold Nanoparticles: A Model Study as Potential Fungicides

Francis J. Osonga [1], Ali Akgul [2], Idris Yazgan [3], Ayfer Akgul [4], Gaddi B. Eshun [1], Laura Sakhaee [3] and Omowunmi A. Sadik [1,*]

[1] Sensors Mechanisms Research and Technology Center (The SMART Center), Chemistry and Environmental Science Department, New Jersey Institute of Technology, University Heights, 161 Warren Street, Newark, NJ 07102, USA; fosonga1@binghamton.edu (F.J.O.); gbe4@njit.edu (G.B.E.);

[2] Department of Sustainable Bioproducts, College of Forest Resources, Mississippi State University, Starkville, MS 39759, USA; aa1116@msstate.edu

[3] Department of Chemistry, Center for Research in Advanced Sensing Technologies & Environmental Sustainability (CREATES), State University of New York at Binghamton, P. O. Box 6000 Binghamton, NY, 13902, USA; iyazgan1@binghamton.edu (I.Y.); lsakhae1@binghamton.edu (L.S.);

[4] Department of Basic Sciences, College of Veterinary Medicine, Mississippi State University, Starkville, MS 39759, USA; aa1625@msstate.edu

* Correspondence: sadik@njit.edu

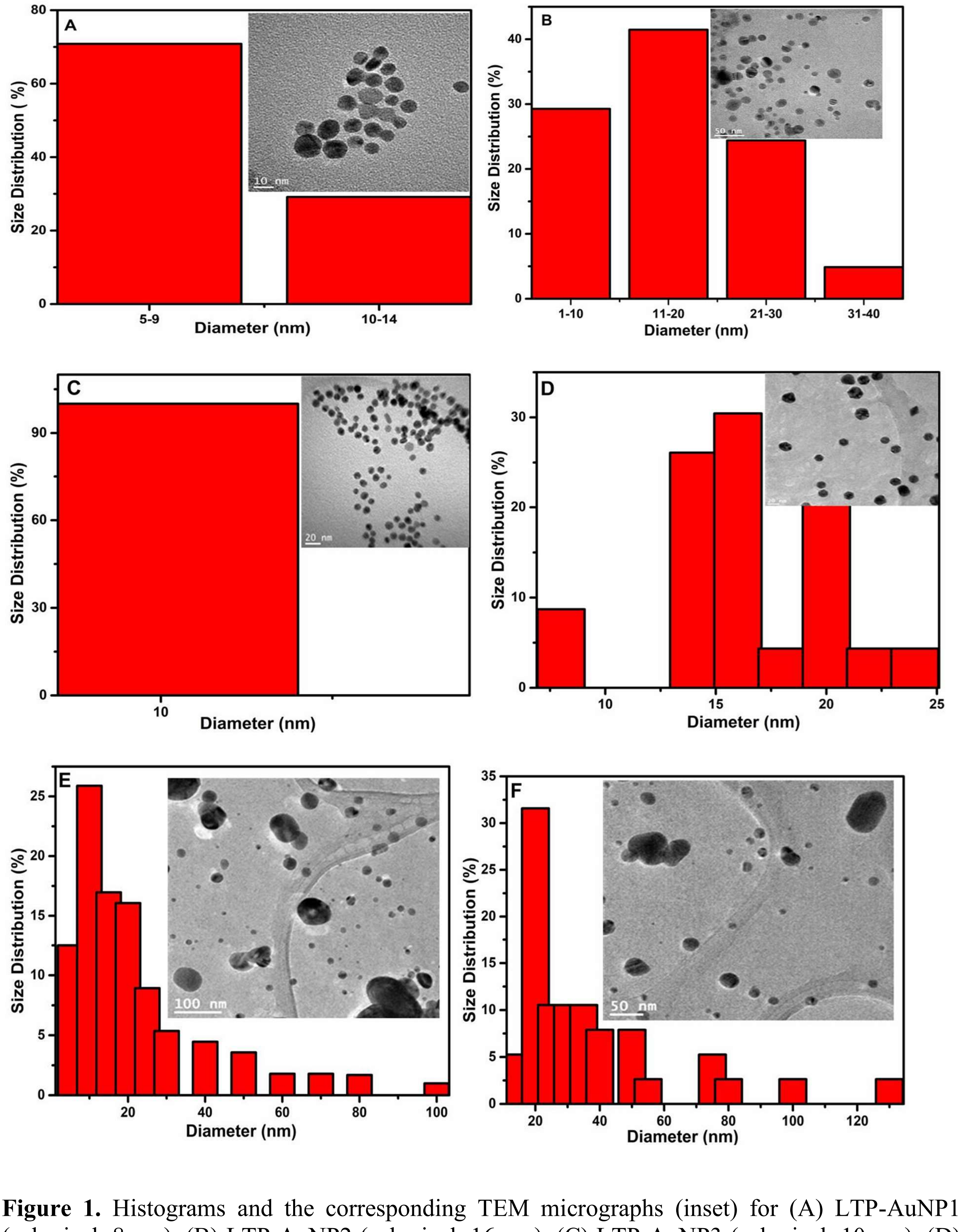

Figure 1. Histograms and the corresponding TEM micrographs (inset) for (A) LTP-AuNP1 (spherical, 8 nm), (B) LTP-AgNP2 (spherical, 16 nm), (C) LTP-AuNP3 (spherical, 10 nm), (D) LTP-AuNP4 (cubic, 16 nm), E) LTP-AgNP5 (quasi-spherical, 21 nm), (F) LTP-AgNP6 (quasi-spherical, 37 nm).

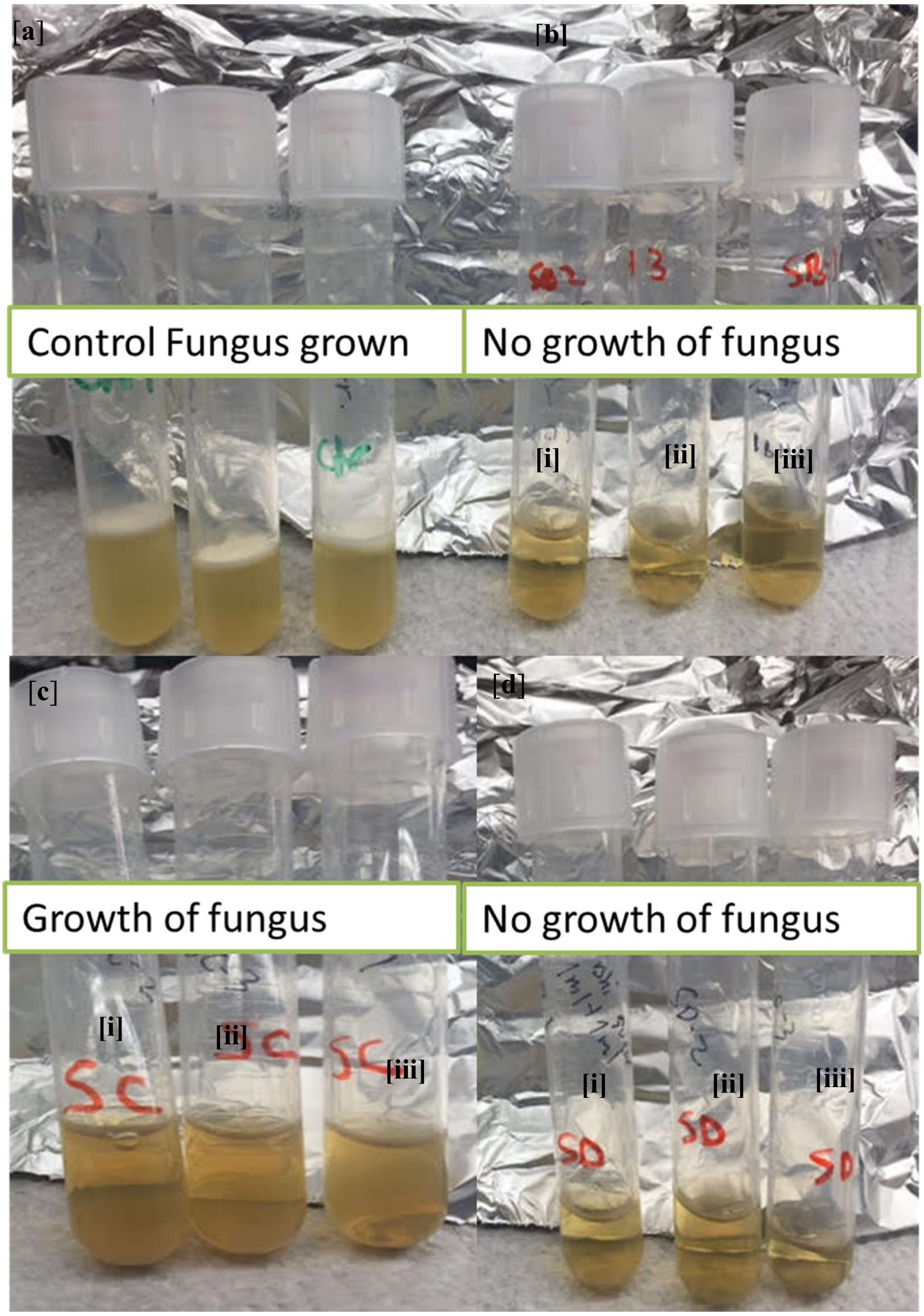

Figure S2: (a) Control of *A.nidulans*, (b) (i) 1 µM, (ii) 2 µM and (iii) 4 µM LTP-AgNP1, (c) (i) 20 µg/mL, (ii) 40 µg/mL and (iii) 80 µg/mL LTP, (d) (i) 36 µM, (ii) 72 µM and (iii) 144 µM LTP-AgNP2 after one week growth.

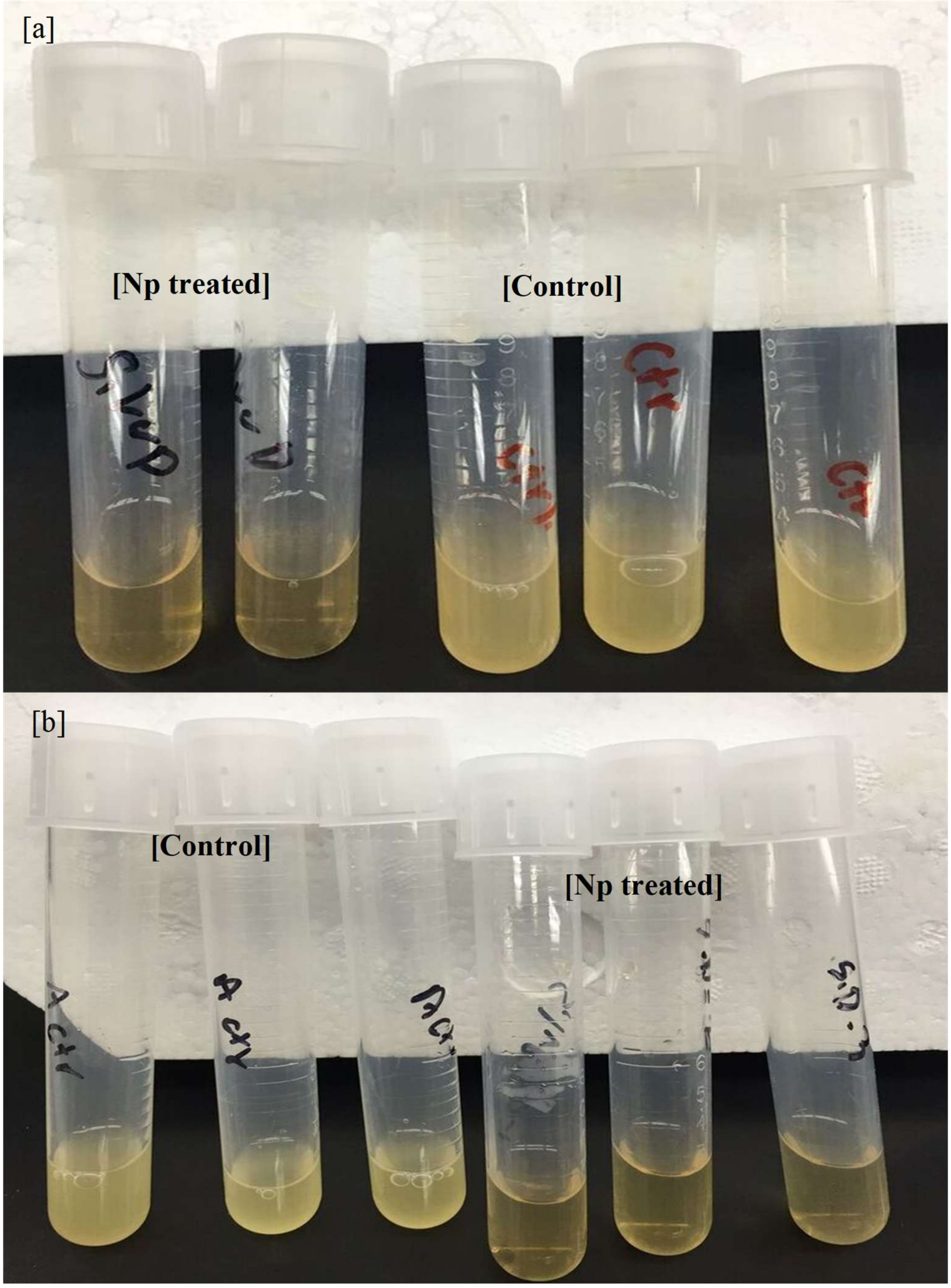

Figure S3: (a) 36 μM LTP-AgNP2 treated *P.aeruginosa*, (b) 18 μM LTP-AgNP2 treated *A.hydrophila*

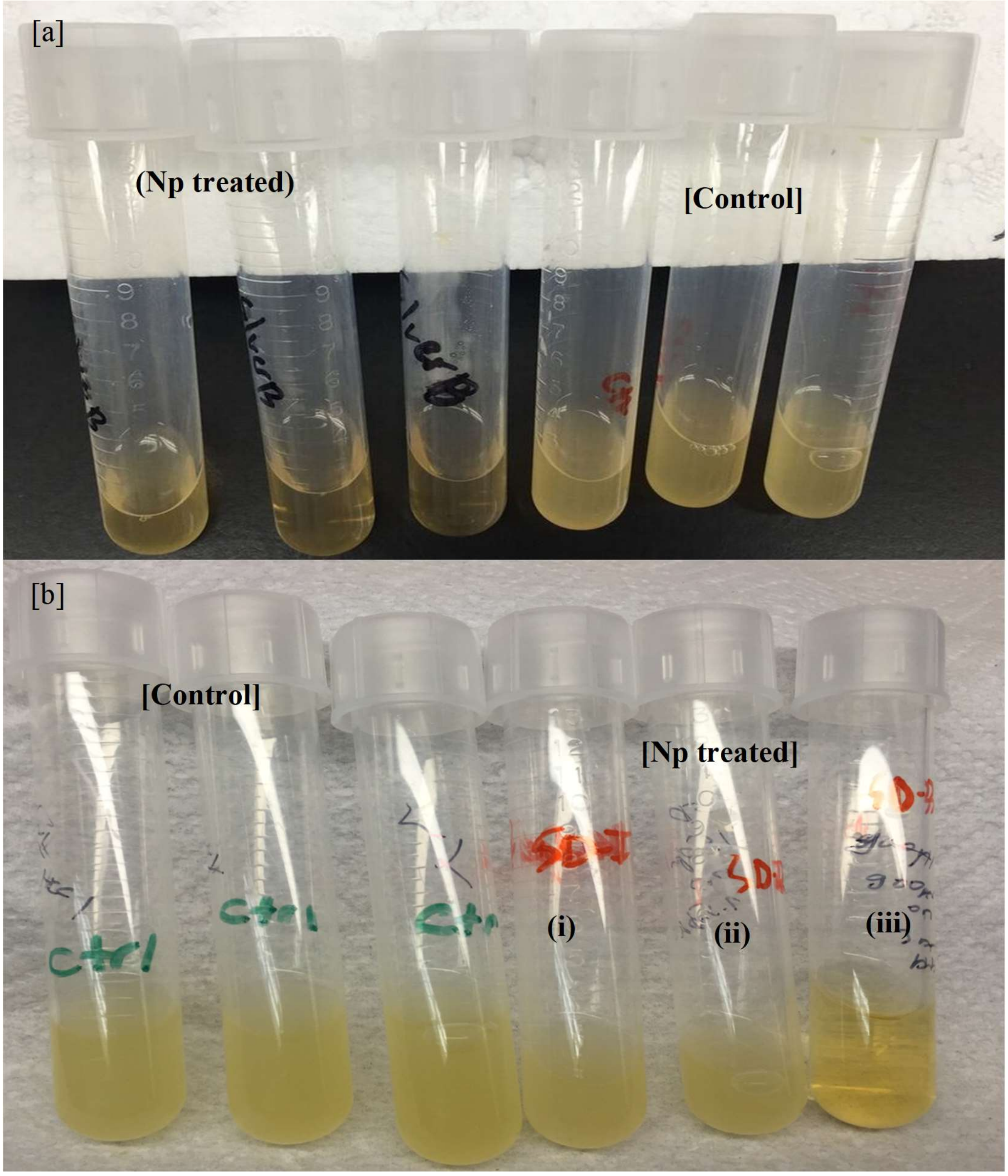

Figure S4: (a) 4 µM LTP-AgNP1 treated *A.hydrophila* and (b) (i) 4.5 µM, (ii) 9 µM and (iii) 18 µM LTP-AgNP2 treated *L.monocytogenes* samples.
